# Moxifloxacin Hydrochloride Electrochemical Detection at Gold Nanoparticles Modified Screen-Printed Electrode

**DOI:** 10.3390/s20102797

**Published:** 2020-05-14

**Authors:** M. Shehata, Amany M. Fekry, Alain Walcarius

**Affiliations:** 1 Chemistry Department, Faculty of Science, Cairo University, Giza 12613, Egypt; mohammed.shehata9011@yahoo.com; 2 LCPME, Université de Lorraine, CNRS, F-54000 Nancy, France

**Keywords:** Moxifloxacin hydrochloride (Moxi), carbon paste electrodes, carbon screen-printed electrodes, gold nanoparticles (AuNPs), modified electrodes, differential pulse voltammetry

## Abstract

It appeared that either the carbon paste or the screen-printed carbon electrodes that were modified with gold nanoparticles (AuNPs) gave rise to the largest current responses after a rapid screening of various nanomaterials as modifiers of carbon composite electrodes in view of designing an electrochemical sensor for Moxifloxacin Hydrochloride (Moxi). The screen-printed electrode (SPE) support was preferred over the carbon paste one for its ability to be used as disposable single-use sensor enabling the circumvention of the problems of surface fouling encountered in the determination of Moxi. The response of AuNPs modified SPE to Moxi was investigated by cyclic voltammetry (CV) (including the effect of the potential scan rate and the pH of the medium), chronoamperometry, and differential pulse voltammetry (DPV) after morphological and physico-chemical characterization. DPV was finally applied to Moxi detection in phosphate buffer at pH 7, giving rise to an accessible concentration window ranging between 8 µM and 0.48 mM, and the detection and quantification limits were established to be 11.6 µM and 38.6 µM, correspondingly. In order to estimate the applicability of Moxi identification scheme in actual trials, it was practiced in a human baby urine sample with excellent recoveries between 99.8 % and 101.6 % and RSDs of 1.1–3.4%, without noticeable interference.

## 1. Introduction

Moxifloxacin Hydrochloride (Moxi) (1-cyclopropyl-6-fluoro-1, 4-dihydro-8-methoxy-7-[(4aS, 7aS)-octahydro-6H-pyrrolo [3, 4-b] pyridin-6-yl] 4-oxo-3 quinoline carboxylic acid) is an antimicrobial fluoroquinolone (antibiotics family) of low toxicity and antimicrobial [1,2] versus bacteria, both gram (+ve) and gram (−ve) [3]. It is mainly mentioned in medical treatments [4,5]. Examining of pharmaceutical molecules, like Moxi, is an important request [6], so that several conventional protocols were operated for this purpose, such as HPLC [7,8], HPLC-UV [9], capillary electrophoresis [10], fluorimetric [11], and chemiluminescence [12]. These techniques were sensitive, but they are usually expensive and time consuming, and they may require quite sophisticated protocols [13]. It is the reason why electrochemical approaches for Moxi detection were proposed as possible alternatives for overcoming these disadvantages, thanks to their simplicity, rapidity, and good analytical performance. Previous examples in the literature are based on various electrodes, like glassy carbon electrode [14], carbon paste electrodes (CPEs) [15,16], and/or molecularly imprinted polymers on electrodes [17].

Heterogeneous carbon-based electrodes (CPEs or carbon screen-printed electrodes, SPEs) and their modified forms are suitable for the determination of different substances due to their fairly inert electrochemistry, negligible background current, easily renewable surface (CPEs), or possible mass production as single-use sensors (SPEs), and simple preparation [18]. The modified CPE or SPE electrodes result in low detection limits, high sensitivities, and selectivity via accumulation or electrocatalysis or both of them [19,20].

In particular, metal nanoparticles and other nanomaterials offer attractive features that can be exploited for electrochemical sensing of biologically-active compounds [21], notably based on modified carbon electrodes. For instance, gold nanoparticles have been utilized in designing various types of sensors, owing to their unique structure and exceptional features, like good biocompatibility, high conductivity, high surface to volume ratio, and adsorption properties [22]. Graphene oxide (GO) has a large surface area, strong mechanical strength, good chemical stability, high conductivity, and excellent catalytic properties due to bearing two-dimensional plane rich with oxygen containing functional groups [23]. GO will be used here with Glutathione, which contains eight active binding sites to act as strong electron donors and antioxidant materials [24,25]. Ag nanoparticles have been utilized in catalysis and electrochemistry due to their great electrical conductivity and stability [26,27]. Ag nanoparticles can be mixed with cellulose acetate phthalate (also utilized in several drug delivery procedures as being nontoxic in human body usages [28]) or chitosan (promising for various biomedical applications [29]) to improve biocompatibility. Nano-Co(II, III) oxides have been significantly applied for electrochemical energy storage devices, electrocatalysis, biosensors, and electrochemically sensing devices [30,31,32,33,34,35,36], because of their high chemical stability, biocompatibility, and huge specific surface areas [37,38,39,40], which permit easy access to a large amount of active sites. Zeolites are promising materials with ion-exchange properties that can be exploited as modifying agents for electrochemical sensors [41,42]. Finally, Carbon Nanotubes (CNTs) exhibit fascinating properties [43,44], such as excellent electronic characteristics, extremely large and active surface areas, and intrinsic electrocatalytic features, which make them unique candidates for electrode modification and sensors development [45,46].

All of these modifiers are possibly interesting for Moxi detection, owing to their preconcentration ability and/or electrocatalytic properties and the first objective of this work was to evaluate them in order to select the most promising one. After a rapid screening by cyclic voltammetry (CV) utilizing carbon composite electrodes modified with the above components, the best system (i.e., based on gold nanoparticles, AuNPs, thanks to their unique structure and high conductivity, high surface to volume ratio, and adsorption properties) was selected and investigated for the electrochemical sensing of Moxi at AuNPs modified screen-printed carbon electrodes. The modified electrodes were examined by various physico-chemical techniques and their electrochemical behavior was characterized by several electrochemical methods prior to Moxi detection by differential pulse voltammetry (DPV). The figures of merit were determined and the best sensors enabled the identification of Moxi in urine samples.

## 2. Materials and Methods

### 2.1. Chemicals and Solutions

The analyte (Moxi) was obtained from the National Organization for Drug Control and Research (Giza, Egypt). The graphite powder and paraffin oil that were utilized to build on the carbon paste (CP) were provided by Merck (Darmstadt, Germany), and the carbon ink used to fabricate the screen-printed electrodes was purchased from Alfa Aesar. Gold nanoparticles were prepared from a gold chloride salt (KAuCl4). Other electrode modifiers were Graphene Oxide (GO) (15–20 sheets, 4–10% edge oxidized), Ag nanoparticles, Nano-Co (II, III) oxide (NCO), CNTs (multi-walled, 3–20 nm OD, 1–3 nm ID, 0.1–10 micron long), l-reduced Glutathione, Cellulose acetate phthalate and chitosan powder (Aldrich, USA), and Zeolite Y (from Alfa Aesar). Britton Robinson (B-R) buffer solutions were prepared from CH_3_COOH, H_3_BO_3_, and H_3_PO_4_ (total concentration: 4.0 × 10^−2^ M), at pH values ranging from 2 to 9 (as adjusted by using 0.2 M NaOH) [47,48]. All of the solutions were prepared utilizing triply distilled water.

### 2.2. Preparation and Modification of Electrodes

#### 2.2.1. Carbon Paste Electrodes (CPEs)

The bare carbon paste electrode (BCPE) was fabricated from mixing 5.0 g of graphite powder with 2.0 ml of paraffin oil into mortar for 10 min. to obtain a homogenous paste. The modified CPEs were prepared through several trials by adding selected amounts of different modifiers to the paste and hand blending the resulting mixtures for 10 min. more and checking their sensitivity toward Moxi in order to achieve the optimum sensor composition in each case. On that basis, 0.05 g of zeolite powder and 0.01 g of CNTs were used to obtain zeolite/carbon nanotubes modified CPE (ZCNTMCPE), 0.05 g of NCO for the nano-Co (II, III) oxide modified CPE (NCOMCPE), 0.05 g of cellulose acetate phthalate powder +0.05 g of chitosan and 0.05 g of silver nanoparticles for the silver nanoparticles/chitosan/cellulose acetate phthalate modified CPE (SCCMCPE), 0.03 g of GO, and 0.05 g reduced glutathione for the graphene oxide/reduced glutathione modified CPE (GORGMCPE). Each CP mixture was then packed into the end of Teflon tube (with diameter of 3 mm, corresponding to 7 mm^2^ electrode surface area) to prepare the corresponding sensors. The excess of composite material was removed and the electrode surface was smoothed while using ultrafine emery paper. Stainless steel ensured the electrical contact. Finally, the gold nanoparticles modified CPE (GNMCPE) was prepared by immersing a BCPE in 0.01 M of gold chloride solution and applying a deposition potential of −0.4 V for 300 s in order to deposit a nano-Au film, which was then air-dried for 5 min. 

#### 2.2.2. Screen-Printed Carbon Electrodes (SPEs)

Screen-printed carbon electrodes (SPEs) were fabricated through ink screen-printing over the ceramic supports, with a silver reference electrode and carbon counter electrode. The PVC insulator was handled to cover the electrodes after being dried for half an hour at 60 °C [49]. BSPE (bare screen-printed electrode) was modified with gold films by means of an electrodeposition method in order to prepare gold nanoparticles modified SPE (GNMSPE). At potential of −0.4 V for 300 s [50], gold film was deposited over SP (screen-printed) carbon surface from 6 mM gold chloride solution (pH = 2.0) and left in air to dry for five minutes in order to be subsequently utilized at the electrochemical measurements. The geometrical surface area of the working electrode was 5 mm^2^.

### 2.3. Methods and Instrumentation 

The electrochemical experiments were carried out using a 25 mL three-electrode cell comprising a platinum auxiliary electrode (CE), the modified CPE or SPE sensors as working electrode (WE), and a saturated calomel electrode (SCE) serving as reference electrode (RE) [51]. Differential Pulse Voltammetry (DPV), Cyclic Voltammetry (CV), Chronoamperometry (CA), and Electrochemical Impedance Spectroscopic (EIS) measurements were performed with the Bio-logic SAS model SP-150 potentiostat monitored by a computer-controlled EC-Lab^®^ electrochemical software. The EIS tests were made at 10 mV ac amplitude in the frequency range of 1.0 mHz to 100 kHz [52] and the equivalent circuit models were built from the EC-Lab^®^ software [50,51,52,53]. Adwa 1030 digitalized pH meter (Romania) that was linked to a glass electrode was used to adjust the solution pH.

The materials and electrodes were characterized by various physico-chemical techniques. Surface morphology was observed by scanning electron microscopy (SEM) while using the Model Quanta 250 Field Emission Gun apparatus coupled with an energy dispersive analysis of X-rays (EDX) unit (FEI Company, Netherlands). Transmission electron microscopy (TEM) analysis was made using a JEM-1400 Electron Microscope (JEOL, Japan). The PAN-analytical X-Ray Diffraction equipment model X′Pert PRO with secondary monochromator, operating at 45 kV and 35 mA with Cu-radiation (λ = 1.542 Å) and scanning speed of 0.04° s^-1^, was used for structural investigations. The diffraction lines located at 2θ values between 2° and 60°, the corresponding spacing (d, Å), and relative intensities (I/I_o_), were obtained. The diffraction charts and relative intensities are obtained and compared with ICDD files.

## 3. Results and Discussion

### 3.1. Preliminary Observations and Surface Characterization of the AuNPs Modified Screen-Printed Electrodes 

**Figure 1:**
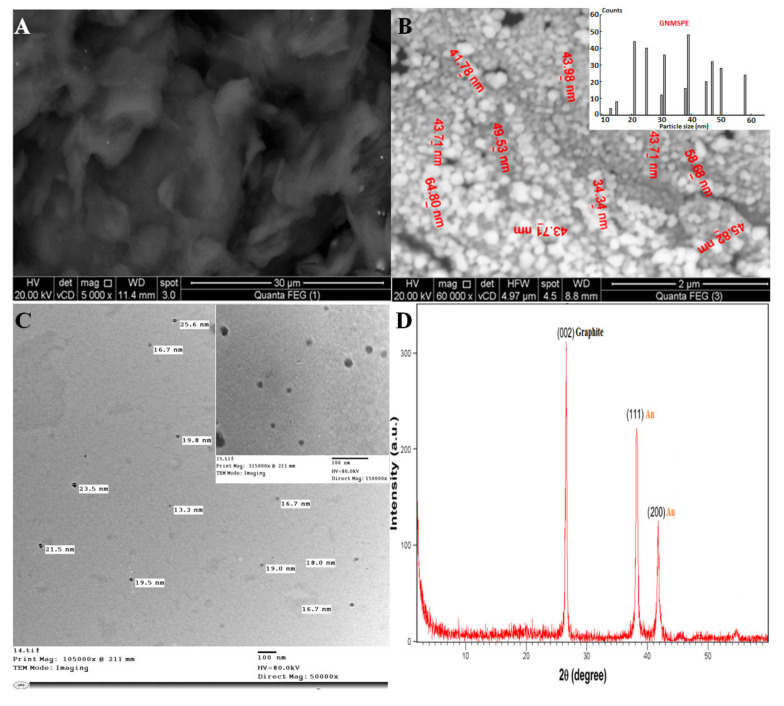
Scanning electron microscopy (SEM) micrographs of the screen-printed electrode surfaces (**A**: screen-printed electrode (SPE) and **B**: gold nanoparticles modified SPE (GNMSPE) accompanied with its histogram); (**C**) transmission electron microscopy (TEM) micrograph of Au nanoparticles; and, (**D**) XRD pattern of GNMSPE.

**Figure A1:**
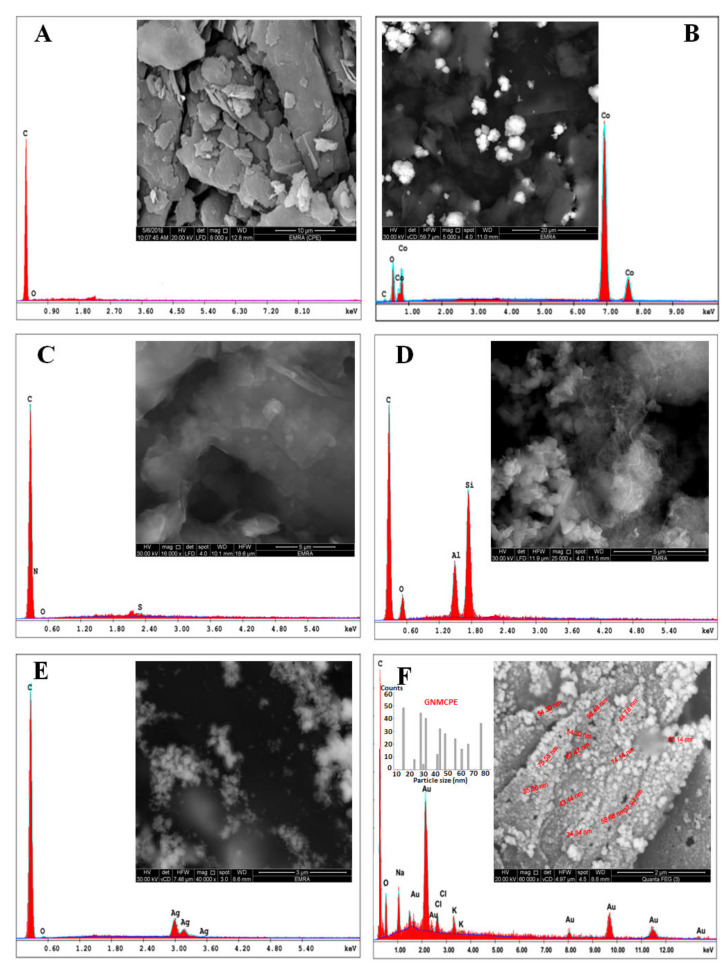
EDX spectra and SEM micrographs (insets) of (**A**) BCPE, (**B**) NCOMCPE, (**C**) GORGMCPE, (**D**) ZCNTMCPE, (**E**) SCCMCPE and (**F**) GNMCPE with its histogram, respectively.

**Figure A2:**
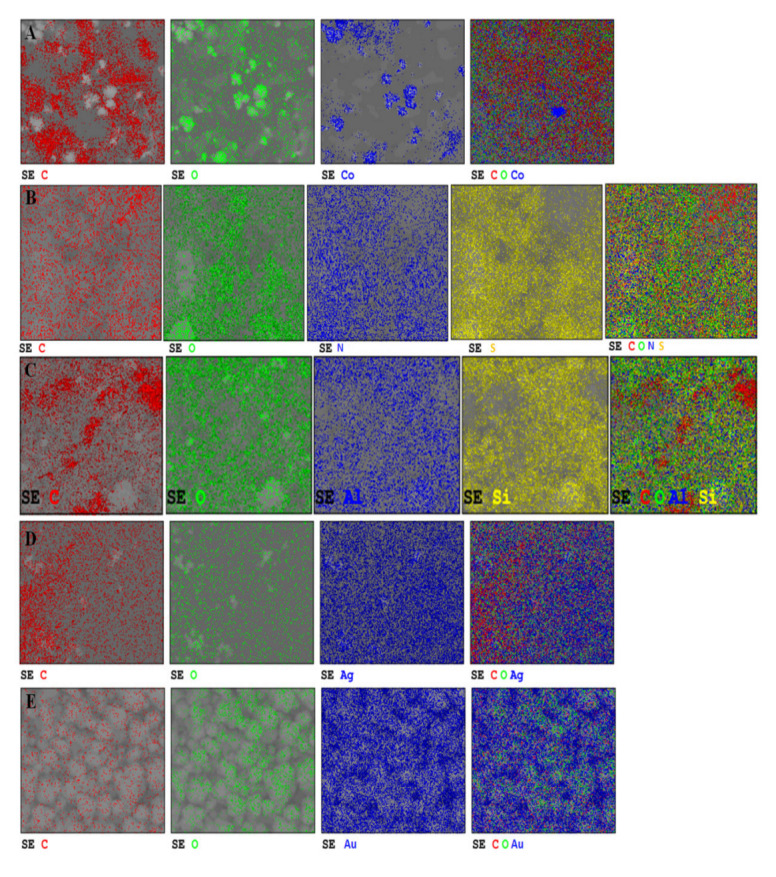
EDX mapping pictures of (**A**) NCOMCPE, (**B**) GORGMCPE, (**C**) ZCNTMCPE, (**D**) SCCMCPE and (**E**) GNMCPE, respectively.

**Figure A3:**
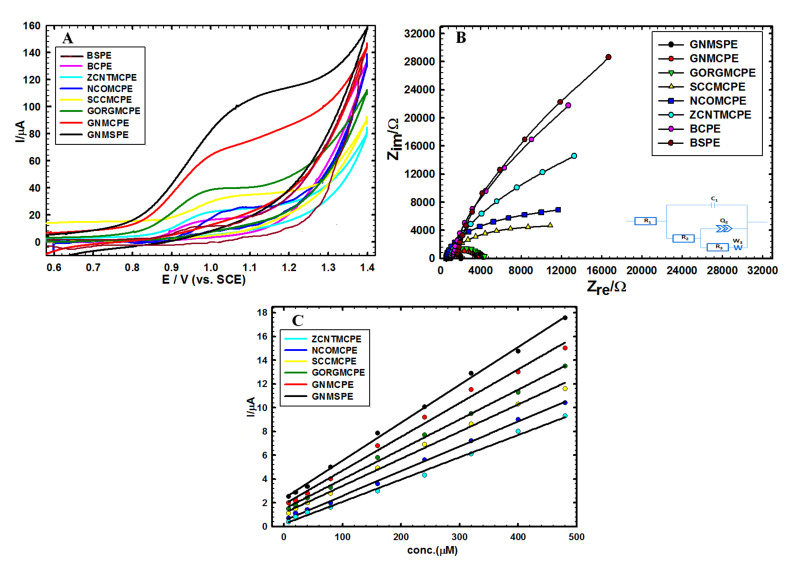
(**A**) CVs of 1.0 mM Moxi in 0.01 M B-R (pH = 7.0) and scan rate 50 mV s^−1^ measured on the various investigated electrodes as presented in the experimental section. (**B**) The corresponding Nyquist plots for the studied sensors (bottom right inset: the best fitted equivalent circuit). (**C**) The analytical curves obtained from performing DPV measurements at scan rate 10 mV s^−1^ with sensing platforms ZCNTMCPE, NCOMCPE, SCCMCPE, GORGMCPE, GNMCPE and GNMSPE.

Carbon paste electrodes modified with various nanomaterials were first prepared and characterized (see Figure A1 and Figure A2 in Appendix A) and screened by cyclic voltammetry as a test to evidence the most appropriate additive to the carbon composite electrode for the electrochemical detection of Moxi (see Figure A3 in Appendix B). When investigated in 1 mM Moxi solution (buffered at pH 7.0), from CV, EIS and calibration data (see respectively parts A, B, and C in Figure A3 in Appendix B), and the best performance (most sensitive detection) was obtained with the composite carbon electrodes covered with gold nanoparticles. The screen-printed electrode modified with gold nanoparticles (GNMSPE) was selected here for the detection of Moxi and its surface characterization was achieved while using various techniques (Figure 1).

SEM, TEM, and XRD characterized GNMSPE (Figure 1). Comparing the SPE surface observed before (Figure 1A) and after gold electrodeposition (Figure 1B) indicates the successful formation of gold nanoparticles well dispersed onto the electrode surface, with spherical shape (and sizes of 13–58 nm, as evaluated from TEM examination, see Figure 1C). As compared to the GNMCPE (Figure A1F), it seems that the density of gold NPs is slightly larger on GNMSPE (Figure 1B). The XRD pattern of the GNMSPE shows sharp diffraction lines at 26.6°, 38.1°, and 42.1° (Figure 1D). The two sharp diffraction lines located at 2θ = 38.1° (with d-spacing of 2.6 Å) and 42.1° (with d-spacing of 2.0 Å) correspond to the Au nanoparticles, while the third one that is located at 2θ = 26.6° (with d-spacing of 3.36 Å) is attributed to carbon, the other main constituent of the modified electrode. No additional lines originating from any other crystalline elements can be noticed, which indicates the high purity of the deposited Au-nanoparticles and that their good crystallinity is supported by the sharpness of diffraction lines. The definite line broadening of the peaks suggests that Au-particles are in the nanometer size range.

### 3.2. Parameters Affecting the Detection, Optimization and Analytical Performance

#### 3.2.1. Influence of Potential Scan Rate

**Figure 2:**
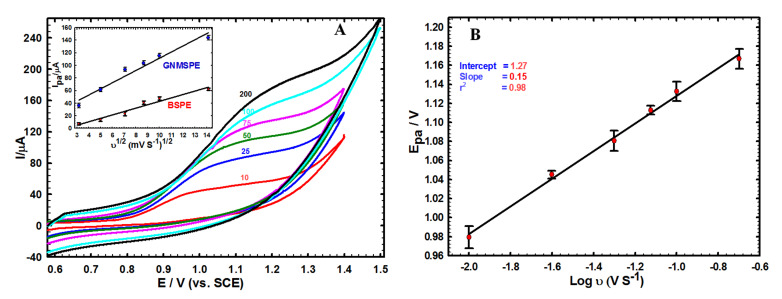
(**A**) Cyclic voltammetry’s (CVs) of 1.0 mM Moxi on GNMSPE recorded at various potential scan rates (v). Inset: variations of anodic peak current (Ipa) with the square root of scan rate (v^1/2^) for BSPE and GNMSPE. (**B**) Variation of the anodic signal potential of GNMSPE with the logarithm of scan rate.

Figure 2 illustrates the effect of potential scan rate on the voltammetric response of Moxi at GNMSPE. The anodic peak currents increased with rising the scan rate (ν = 10–200 mV/s, see Figure 2A), giving a linear variation with respect to the square root of scan rate (see inset in Figure 2A). The linear regression equations for both the bare and GNMSPE electrodes are respectively: Ip (μA) = −10.66 + 5.39 ν1/2 (mV s^−1^) (r_2_ = 0.956) and Ip (μA) = 14.40 + 9.75 ν1/2 (mV s^−1^) (r_2_ = 0.960). This indicates that the oxidation process of Moxi is under diffusion control. It is also noticeable that the Moxi signals are shifted to the positive direction while increasing the potential scan rates (Figure 2B), confirming the irreversibility of the electron transfer reaction [54].

#### 3.2.2. pH Effect and Sensor Stability 

**Figure 3:**
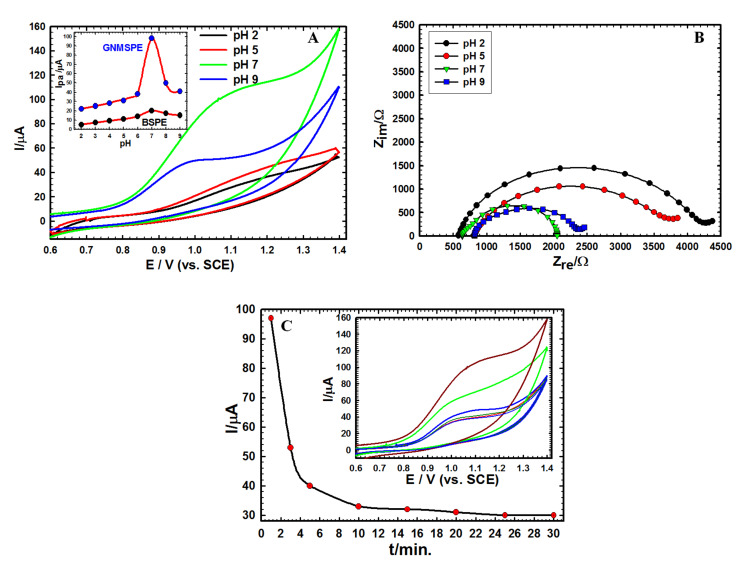
(**A**) CVs of 1.0 mM Moxi on GNMSPE in 0.01 M B-R buffer with different pH values at scan rate 50 mV s^−1^. Inset: variation of anodic peak current (I_pa_) for BSPE and GNMSPE as function of pH. (**B**) Nyquist plots of Moxi on GNMSPE at different pH values. (**C**) Variation of anodic peak current (I_pa_) obtained for successive analyses at increasing times (1–30 min.) using the same GNMSPE in B–R buffer pH 7.0 containing 1.0 mM Moxi. Inset: the corresponding CVs.

The effect of pH on Moxi oxidation was evaluated in B-R buffer (pH 2.0–9.0) at BSPE and GNMSPE, respectively (Figure 3A). The optimum pH corresponding to the largest currents was 7.0 for both electrodes, but the signal intensity was much larger on the modified electrode (in agreement with the results of Figure A3A in Appendix B). The best performance at neutral pH was also evidenced from impedance data (Figure 3B). Though not easily measurable due to a peak-to-wave shape, the voltammetric signals shifted to lower potential values at higher pH, with a slope change around pH 6, consistent with the Moxi pK_a_ value of 6.25 [55]. Such behavior also agrees with the transfer of protons that are involved in the electrochemical oxidation of structurally related compounds [56].

The stability of GNMSPE was tested in B-R buffer solution comprising 1 mM of the analyte through CV technique (Figure 3C). It was demonstrated that the signal intensity decreased rapidly with lengthening the immersion time of sensor: starting around 100 µA, the signal intensity dropped by one half of its value after only one more CV cycle, to level down to about one-third of its initial value after some minutes of successive analyses. This is probably due to the irreversible adsorption of the Moxi oxidation products onto the electrode surface and/or the poisoning of the surface active sites (Au nanoparticles) by chelation with Moxi [15,57], justifying the need for a single-use sensor, as GNMSPE, thanks to the screen-printing technology to produce such electrodes.

#### 3.2.3. Chronoamperometry 

**Figure 4:**
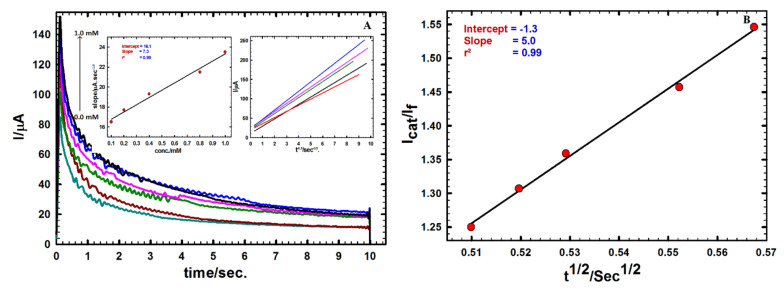
(**A**) Chronoamperograms at GNMSPE for different concentration of Moxi (0.0–1.0 mM). Insets: Variations of I vs. t^−1/2^ built from chronoamperograms (right) ant plot of the corresponding slopes against Moxi concentration (left). (**B**) Variation of I_cat_/I_f_ ratio versus t^1/2^.

Figure 4A shows chronoamperograms that were recorded at a constant potential of +1.03 V vs. SCE utilizing GNMSPE in BR buffer (pH = 7.0) containing various Moxi concentrations.

Utilizing the Cottrell Equation (1), the diffusion coefficient (D) can be evaluated from plotting I versus t^−1/2^ [58] (see right inset in Figure 4A).

(1) $$i=\frac{nFAC^{o}\sqrt{D}}{\sqrt{\pi t}}$$

where i = current, in unit A, F = Faraday constant (96485 C/mol), n = number of electrons, A = area of the electrode in cm^2^, c^0^ = bulk concentration of the analyte in mol/cm^3^; t = time in s, and D = diffusion coefficient for species in cm^2^/s.

A calibration curve can be obtained as a linear relationship between different Moxi concentrations (0 to 1.0 mM) and the slopes obtained from the current relation versus t^−1/2^ (Figure 4A, left inset), leading to the calculation of the diffusion coefficient, D value for Moxi equal to 3.57 × 10^−6^ cm^2^ s^−1^. The catalytic rate constant k_cat_ (mol^–1^ L^–2^ s^−1^) was obtained from the relation (2) [59]:

(2) IcatIf=πkcatCot12

with I_cat_/I_f_ being the ratio between the currents sampled in the presence (I_cat_) or absence of Moxi (If), C_0_ the bulk concentration of Moxi (mol L^−1^), and t the elapsed time (s).

The mean catalytic rate constant was evaluated from the slope of I_cat_/I_f_ versus t^1/2^ plot (Figure 4B), and a value of k_cat_ = 7.91 × 10^3^ mol^−1^ L^−2^ s^−1^ was calculated for a Moxi concentration of 1.0 mM. The high K_cat_ value obtained indicates quite fast charge transfer processes between the analyte and the modified electrode surface and supports the interest of AuNPs for Moxi determination.

### 3.3. Calibration Plot and Detection Limit

**Figure 5:**
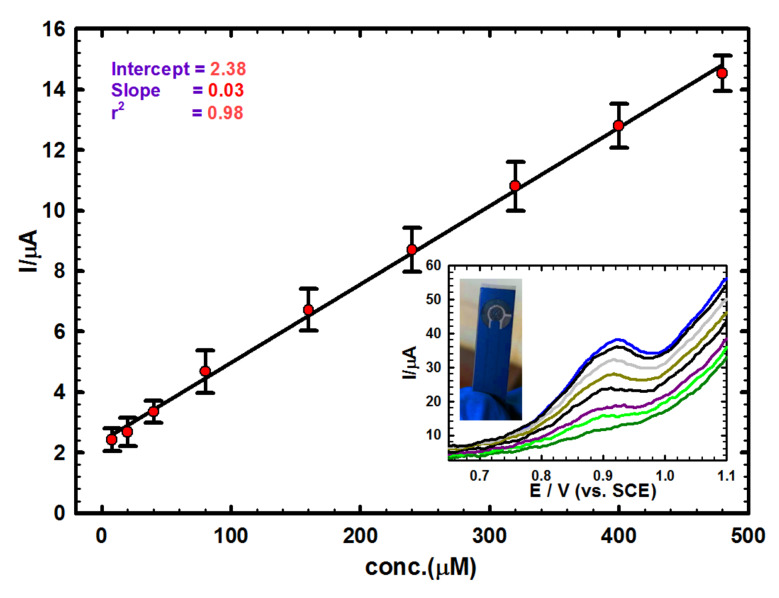
Calibration plot for Moxi, Inset: corresponding differential pulse voltammetry’s (DPVs) for successive addition of Moxi in B-R buffer solution of pH 7.0 at scan rate = 10 mV s^−1^ using GNMSPE and illustrative picture of a typical SPE used in this work.

Differential pulse voltammetry (DPV) was used as sensitive technique for building the calibration plot for Moxi (Figure 5). As shown, a linear variation of peak currents with Moxi concentration was obtained in the 8.0–480 µM range (in B-R buffer, pH 7.0): Ip (µA)= 2.38 + 0.026 C (µM), r^2^ = 0.984. The limit of detection (LOD = 3 σ/m) and limit of quantification (LOQ = 10 σ/m), where, σ is the standard deviation and m is the calibration plot slope were obtained as 11.6 µM and 38.6 µM, respectively. Comparing the obtained data for Moxi determination by GNMSPE against others analytical or methods [7,60,61] or electrochemical techniques [12,13,14] shows acceptable performance of the proposed novel sensor, offering opportunities for on-site analyses thanks to the possible mass production of low-cost screen-printed electrodes and the suggested electrochemical sensor for Moxi detection also compared with others [17,62,63,64,65,66,67] in terms of detection limits, as shown in Table 1.

### 3.4. Real Sample Analysis, Interfering Species and Simultaneous Determination Test

**Figure 6:**
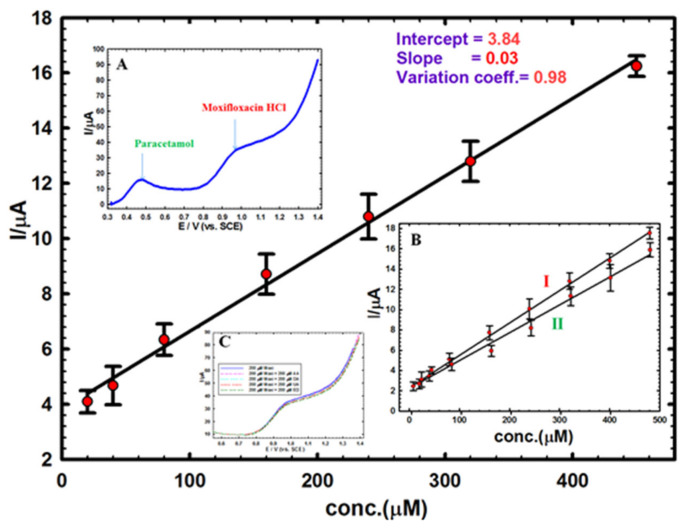
Calibration plot of Moxi in a urine sample prepared in B-R buffer solution (pH 7.0) using GNMSPE. Inset A: separate detection of 200 µM Moxi with 200 µM paracetamol by DPV in the same solution. Inset B: I) calibration plot for Moxi in the presence of 200 µM paracetamol, and II) calibration for paracetamol in the presence of 200 µM Moxi. Inset C: detection of 200 µM Moxi alone or in the presence of 200 µM ascorbic acid, dopamine, uric acid or estradiol).

A calibration plot was established utilizing the GNMSCPE in a diluted urine medium, giving a linear correlation over a concentration range of 20–450 µM, in order estimate the method applicability to real sample analysis (Figure 6). The linear regression equation that was obtained from the variation of DPV peak currents as a function of the Moxi concentration s was: Ipa (µA) = 0.028C (µM) + 3.84, r^2^ = 0.98). The LOD (3σ) was 10.7 µM, and the LOQ (10σ) was 35.6 µM. Four different concentrations were chosen on the calibration plot through standard addition method for the examination of Moxi in buffered solution of pH 7.0, and each measurement was repeated five times in order to evaluate the accuracy and precision of the approach, as represented in Table 2. The detected concentrations of Moxi were always very near to the expected values, giving acceptable recoveries for all samples between 99.8 % and 101.6 % with RSDs of 1.1–3.4 %.

In order to investigate the interference effects for other compounds that can co-exist with Moxi in urine samples such as uric acid (UA), ascorbic acid (AA), dopamine (DA) and hormones like estradiol (ED), a fixed amount of Moxi (200 µM) spiked with the same concentration of these substrates and evaluated under the same experimental conditions through DPV. The results (see bottom inset C in Figure 6) indicate the negligible effect of interfering substrates (i.e., 0.8%, 1.8%, 3.7%, and 5.2% signal intensity decrease for ascorbic acid, dopamine, uric acid, and estradiol, respectively), which ensures good sensor selectivity. However, it can be anticipated that structurally related fluoroquinolones could interfere and the sensor would be not selective to Moxi in this case.

Typically, a patient suffering from dry cough and high fever rate should receive 200 mg moxifloxacin orally each half a day and 500 mg paracetamol when necessary [68], justifying the need for simultaneous detection. Hence, the immediate identification of 200 µM Moxi with 200 µM paracetamol in pH 7.0 utilizing GNMSPE was achieved (see top left inset A in Figure 6). The curve demonstrates a sufficient separation between the two signals, i.e. paracetamol (at 0.49 V) and Moxi (at 0.98 V), with ΔE of 0.49 V, enabling the possible simultaneous detection of both Moxi and paracetamol in the same medium. To support these findings, a calibration for Moxi in the presence of a constant concentration of paracetamol and, the opposite, a calibration for paracetamol in the presence of a constant concentration of Moxi, were made (see right bottom inset B in Figure 6), confirming the interest of GNMSPE for the simultaneous detection of these two pharmaceuticals.

### 3.5. Reproducibility and Long-Term Stability

Five distinct electrodes were tested for the voltammetric determination of 50 µM Moxi without obvious dispersion of the data (RSD value of 2.3%), which ensures a preparation process highly trustworthy, in order to validate the reproducibility of GNMSPE in terms of RSD.

The long-term stability of the proposed electrode was evaluated through storing GNMSPE in a refrigerator (4 ℃) for seven days. After that, a single voltammetric measurement for Moxi was carried out, revealing a current response at 95% of the value measured directly after fresh preparation, indicating good storage stability of the electrode.

## 4. Conclusions

This work brings together a comparison between several modifiers of heterogeneous carbon electrodes, pointing out the interest of gold nanoparticles on carbon paste or screen-printed carbon electrodes for the electrochemical sensing of Moxi. SPE that was modified with simple electrochemically deposited gold nanoparticles, GNMSPE, showed a high sensitivity and applicability for detecting Moxi in aqueous solutions and urine samples under physiological conditions with acceptable LOD (11.6 µM), wide linear concentration range (8–480 µM), and excellent recoveries (ranging between 99.8% and 101.6%) and RSDs (1.1–3.4%, n = 5). Sensor optimization was achieved from data that were collected with the aid of numerous electrochemical techniques (CV, DPV, EIS, and CA). The GNMSPE electrochemical sensor can be also applied to Moxi determination in the presence of paracetamol.

## Figures and Tables

**Table 1:** Comparison of the analytical figures of merit for the present method with others approaches for the determination of Moxi.

The System	Technique	LOD (M)	Linear Range (M)	Ref
Molecularly imprinted polymer modified carbon paste electrode	Electrochemical	5.9 × 10^−8^	3.13 × 10^−6^–2.0 × 10^−4^	[17]
Interaction study of moxifloxacin with Cu(II) ion	Square-wave voltammetry	3.6 × 10^−8^	0.1 × 10^−6^–3.5 × 10^−6^	[62]
Bifunctional monomer molecularly imprinted film at graphene modified glassy carbon electrode	Voltammetric	5.12 × 10^−10^	1.0 × 10^−9^–5.0 × 10^−5^	[63]
Carbon paste modified with silver nanoparticles	Electrochemical	2.9 × 10^−9^	7.0 × 10^−7^–1.8 × 10^−4^	[15]
Molecularly imprinted polymer	Potentiometric based PVC membranes	1.7 × 10^−6^	1.0 × 10^–5^–1.0 × 10^−2^	[64]
New coated platinum selective electrode	Potentiometric titration	9.2 × 10^−6^	1.0 × 10^-5^–1.0 × 10^−3^	[65]
A green HPLC assay method	HPLC	0.23 µg/mL	2.0–40.0 µg/mL	[66]
molecularly imprinted polymer	PLC-MS/MS method	0.03 µg/mL	0.2–1.2 µg/mL	[67]
GNMSPE	Electrochemical	11.6 × 10^−6^	8 × 10^−6^–0.48 × 10^−3^	This work

**Table 2:** Recovery and standard deviation data for Moxi detection in urine sample with GNMSPE.

Spiked Moxi (µM)	Detected Moxi ^1^ (µM)	Av. Recovery ^1^ (%)	RSD ^2^ (%)
25.0	25.4	101.6	1.1
75.0	74.9	99.8	3.4
150.0	149.8	99.9	1.9
300.0	300.6	100.2	2.6

^1^ Average values from 5 independent measurements (n = 5). ^2^ Relative Standard Deviation.

## Appendix A

### Surface Description of the Modified Carbon Paste Electrodes

The SEM micrograph of the bare carbon paste electrode (BCPE) reveals a rather rough surface with EDX confirming the presence of carbon and trace of oxygen atoms (Figure A1A). Data obtained for cobalt oxide nanoparticles (<100 nm) modified carbon paste electrode (NCOMCPE) and corresponding EDX analysis confirm the additional existence of cobalt in the form of well dispersed ice-like shaped particles on the electrode surface (Figure A1B). The micrograph obtained for GORGMCPE sensor and the associated EDX data endorsing the existence of S and large carbon content (Figure A1C) are consistent with dispersed graphene oxide blended with reduced glutathione of average size less than 445 nm. From the micrograph recorded for the ZCNTMCPE sensor, which comprises a zeolite and multi-walled carbon nanotubes (3–20 nm OD, 1–3 nm ID, 0.1–10 µm long), one can distinguish (Figure A1D) the zeolite crystals along with dispersed carbon nanotubes bundles blended with the aluminosilicate particles (also evidenced from EDX confirming significant amounts of Si, Al and O). The SCCMCPE sensor contains AgNPs, chitosan and cellulose phthalate on the CPE surface, which agrees with both EDX data proving the existence of silver and SEM showing dispersed Ag nanoparticles with an average size of 50 nm (Figure A1E). Finally, the observation of GNMCPE confirms that gold nanoparticles have been successfully deposited in a homogenous pattern onto the carbon paste surface (Figure A1F) with a size of 14–75 nm.

EDX mapping of all the above electrode surfaces (Figure A2) supports the quite good uniformity of all blended substances in the carbon paste matrix although it is not possible to distinguish all individual components/modifiers by this way.

## Appendix B

### Voltammetric Response of Moxifloxacin Hydrochloride at the Various Electrodes

Cyclic voltammetry was first used as a screening test to evidence the most appropriate additive to the carbon composite electrode, and this has been investigated in 1 mM Moxi solution (buffered at pH 7.0) at a potential scan rate of 50 mV s^−1^ (Figure A3A). Because of ease of preparation and modification, the carbon paste electrodes were used for such screening and then the best system was extended to the screen-printed electrode, for further studies and practical application. As shown, an oxidation peak for Moxi appeared around 1.0 V and its intensity followed a trend (GNMCPE > GORGMCPE > SCCMCPE > NCOMCPE > BCPE> BSPE, with respective current values of 58.1 > 23.8 > 18.0 > 17.4 > 16.0 > 13.1 > 12.8 µA) indicating that the composite electrode covered with gold nanoparticles led to the most sensitive detection. The performance of gold nanoparticles deposited onto the screen-printed electrode was even better (signal current as high as 97.0 µA for GNMSPE, i.e. almost one order of magnitude larger than the response of an unmodified carbon electrode). This can be accredited to the enhanced conductivity brought by the gold nanoparticles but also to the possible chelation of Moxi to the gold surface (i.e., through the electron pairs on the hydroxyl (oxygen atoms) and/or amide groups (nitrogen atoms) or through the resonance of the double bonds on the six carbon atoms rings [17], as previously reported for Moxi binding to silver nanoparticles [15,57].

The above trend in current response was also consistent with impedance data (see Nyquist plots in Figure 3B). The best model [69,70,71] that suits the measurements (Figure A3B, inset) is a two-time constants model (parallel) comprising R_1_ (solution resistance), R_2_, R_3_ (Charge transfer resistance), W (Warburg impedance), C_1_ (capacitance), and Q_2_ is a constant phase element of capacitance (representing surface non-ideality). Hence, the reaction model is contingent on both of diffusion and charge transfer processes [72]. From data fitting of Nyquist plots according to this equivalent circuit model, one can evaluate impedance values of ca. 1.9 KΩ (highly conductive) for GNMSPE, 20 KΩ for the bare CPE and 28 KΩ for the bare SPE (lowest conductivity) for the bare CPE. This confirms the choice of GNMSPE as the most appropriate sensor for the present work, which will thus be used exclusively afterwards owing to its highest current and conductivity. In order to prove our hypothesis and ensure the enhanced conductivity brought by the gold nanoparticles, we performed one EIS measurement before CV of the Figure 3A using BSPE (showing high resistance) and one EIS measurement after CV using GNSPE (showing low resistance) as represented by Figure A3B.

Figure A3C represents a comparison between the analytical curves obtained from performing DPV measurements at scan rate 10 mV s^−1^ with sensing platforms ZCNTMCPE, NCOMCPE, SCCMCPE, GORGMCPE, GNMCPE and GNMSPE. As represented in this curve, there are a distinctive difference in the slope of each line owing to the type and amount of each used modifying agent at each sensing platform. The steeper line will represent low conductivity for Moxi by this sensor.
